# Predicting deliverability of volumetric‐modulated arc therapy (VMAT) plans using aperture complexity analysis

**DOI:** 10.1120/jacmp.v17i4.6241

**Published:** 2016-07-08

**Authors:** Kelly C. Younge, Don Roberts, Lindsay A. Janes, Carlos Anderson, Jean M. Moran, Martha M. Matuszak

**Affiliations:** ^1^ Department of Radiation Oncology University of Michigan Ann Arbor MI

**Keywords:** VMAT, optimization, complexity metric, patient‐specific QA

## Abstract

The purpose of this study was to evaluate the ability of an aperture complexity metric for volumetric‐modulated arc therapy (VMAT) plans to predict plan delivery accuracy. We developed a complexity analysis tool as a plug‐in script to Varian's Eclipse treatment planning system. This script reports the modulation of plans, arcs, and individual control points for VMAT plans using a previously developed complexity metric. The calculated complexities are compared to that of 649 VMAT plans previously treated at our institution from 2013 to mid‐2015. We used the VMAT quality assurance (QA) results from the 649 treated plans, plus 62 plans that failed pretreatment QA, to validate the ability of the complexity metric to predict plan deliverability. We used a receiver operating characteristic (ROC) analysis to determine an appropriate complexity threshold value above which a plan should be considered for reoptimization before it moves further through our planning workflow. The average complexity metric for the 649 treated plans analyzed with the script was $0.132\;{\text{mm}}^{-1}$ with a standard deviation of $0.036\;{\text{mm}}^{-1}$. We found that when using a threshold complexity value of $0.180\;{\text{mm}}^{-1}$, the true positive rate for correctly identifying plans that failed QA was 44%, and the false‐positive rate was 7%. Used clinically with this threshold, the script can identify overly modulated plans and thus prevent a significant portion of QA failures. Reducing VMAT plan complexity has a number of important clinical benefits, including improving plan deliverability and reducing treatment time. Use of the complexity metric during both the planning and QA processes can reduce the number of QA failures and improve the quality of VMAT plans used for treatment.

PACS number(s): 87.55.de, 87.55.Qr, 87.56.jk

## I. INTRODUCTION

The inverse optimization of volumetric‐modulated arc therapy (VMAT) treatment plans has a highly degenerate solution space, meaning that there are a multitude of plan designs that produce very similar calculated dose distributions. These plans can range from simple (low monitor units (MU), low degree of modulation) to highly complex (high MU, high degree of modulation). Simpler plans are preferable for a number of reasons. Highly modulated plans suffer from a reduced confidence in the dose calculation accuracy and a greater dependence on the accuracy of multileaf collimator (MLC) leaf positioning and modeling in the treatment planning system.^1^, ^2^ A greater number of MU leads to increased inter and intra‐MLC leaf leakage dose, treatment time, and susceptibility to motion and interplay effects.^(3)^ Finally, the increased delivery time

of highly modulated VMAT plans (primarily an issue with hypofractionated delivery) partially defeats one of the main advantages of VMAT over IMRT: faster delivery.^(4^)

Several studies have focused on developing plan complexity metrics to correlate with delivery accuracy for both IMRT and VMAT^(57)^ A study that showed how many of these metrics are able predict individual static aperture dose calculation accuracy, including the metric developed at our institution and used in the current work, was recently performed by Götstedt et al.^(8)^ In our previous work,^(9)^ we used this metric to add a plan's aperture complexity as a penalty in the inverse optimization process within an in‐house treatment planning system. We found that the generated plans were simpler and more accurately deliverable with very similar dosimetric characteristics compared to the considerably more complex plans generated without the penalty. At present, there are no commercial treatment planning systems that allow this kind of penalization during optimization. Additionally, the user has few ways to reliably quantify plan complexity, needing to depend primarily on easily calculable metrics such as MU/Gy or subjective measures such as visual inspection of the optimized MLC leaf sequences.

The primary purpose of this work is to determine whether our aperture complexity metric for VMAT plans can predict a plan's deliverability when applied post‐optimization in a commercial treatment planning system. To that end, we developed a software tool that quantifies plan complexity using the metric to identify plans that are unnecessarily complex. Such plans may be chosen to be reoptimized prior to moving further along the plan‐preparation workflow. The tool we have developed compares plan complexity to previously optimized treatment plans that are known to be deliverable (i.e., have passed rigorous pretreatment quality assurance). In this work, we describe the plan complexity metric, some useful features of the software tool, and quantify the metric's ability to successfully predict plan deliverability for a range of treatment sites.

## II. MATERIALS AND METHODS

### A. Complexity metric

The complexity metric used here was described in detail in Younge et al.^(9^) Here we briefly describe how the complexity metric is calculated per control point aperture, per arc, and per plan. The plan metric is defined as

(1) $$M=\frac{1}{MU}\sum_{i=1}^{N}MU_{i}\times \frac{y_{i}}{A_{i}}$$

where the sum is over all control point apertures from I=1 to *N, MU* is the total number of MU in the plan, $MU_{i}$ is the number of MU delivered through aperture $i,A_{i}$ is the open area of aperture *i*, and $y_{i}$ is the aperture perimeter excluding the MLC leaf ends. The metric for a single arc sums over only the apertures in that arc, and MU becomes the arc MU. The metric for a single aperture is simply y/A. The design of the plan complexity metric makes it independent of the plan dose and relatively insensitive to the magnitude of the treatment volume. This design permits a comparison of plan complexities over a wide variety of plan types.

### B. Complexity script

**Figure 1:**
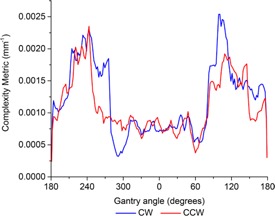
Complexity metric per control point as a function of gantry angle for a head and neck VMAT plan with clockwise (CW) and counterclockwise (CCW) arcs.

We implemented a complexity analysis tool as a software plug‐in, or script, for Varian's Eclipse treatment planning system (Varian Medical Systems, Palo Alto, CA) using their scripting application programming interface (API) in version 11.0.^(10)^ The script reads the MLC positions of all apertures from the current patient, then calculates and displays the complexity metric of each treatment plan, each arc per plan, and each control point per arc. In addition, the complexity of each control point is plotted as a function of gantry angle (Fig. 1).

Visualization of the complexity metric per control point allows users to quickly see how the complexity varies as a function of gantry angle across each arc. For example, in Fig. 1, there is less modulation toward the anterior and increased complexity toward some posterior gantry angles. By knowing which apertures have high complexity, the user may be able to determine which optimization structures are causing the optimizer to increase plan modulation.

**Figure 2:**
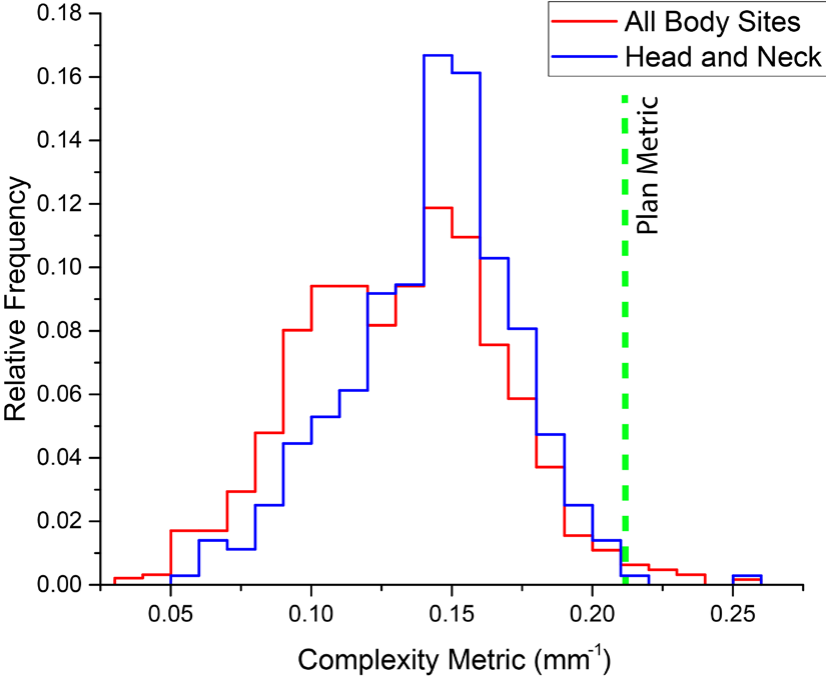
Distribution of complexity metrics for all plans (red) and head and neck plans only (blue). The green dashed line corresponds to the complexity metric of the plan being analyzed by the script. The plan shown here corresponds to the same plan as in Fig. 1.

The script also displays the complexity metric of the current plan in relation to a histogram of complexity data from clinical VMAT plans that passed pretreatment quality assurance (QA) (Fig. 2). These data may be displayed for all plans regardless of treatment site or filtered to a single site. The complexity metric of the plan being analyzed is shown on the histogram to demonstrate whether the plan falls within the standard complexity range.

### C. Complexity threshold

To help predict whether a plan will be deliverable, it is useful to have a single complexity threshold value above which reoptimization should be strongly considered. To determine an appropriate value, we performed a receiver operating characteristics (ROC) analysis using 711 VMAT plans that had already been through the QA process at our institution between 2013 and mid‐2015. A plan that has a complexity metric above the threshold and fails pretreatment QA is a true‐positive. A plan that has a complexity metric above the threshold but passes QA is a false‐positive. It should be noted that there is no single “correct” metric threshold, as any choice will involve a tradeoff between true‐positives and false‐positives. In clinical practice each plan must be evaluated on a case‐by‐case basis. The ROC curve data may also be used to provide the treatment team with the quantitative likelihood of QA failure based on past data. Such an evaluation further strengthens the use of this quantitative tool in clinical practice.

### D. VMAT pretreatment quality assurance

At our institution, pretreatment measurement QA is performed for all VMAT plans prior to treatment delivery using a helical diode array (Sun Nuclear's ArcCHECK phantom, Sun Nuclear Corp., Melbourne, FL) with a Wellhöfer IC‐10 chamber (Scanditronix Wellhöfer North America, Bartlett, TN) positioned within the core of the phantom. VMAT plans are recalculated on this phantom using a 1 mm grid. We require that 95% of measured points pass a composite analysis using 4%/1 mm agreement criteria. We use a global normalization and a 10% dose threshold for analysis. The ion chamber point measurement must be within 4%. These criteria were chosen based on our institution's experience with gradient compensation^11^, ^12^ (Note that we did not use any gradient compensation in this study because it was not available for clinical use. We performed a composite analysis, as opposed to a gamma analysis, which is the most similar option to gradient compensation.) For this study, we used 711 VMAT plans generated at our institution between 2013 and mid‐2015. This included 649 plans that passed QA and 62 plans that failed QA. In this work, all 62 plans that failed QA had failing diode array measurements, and only one of the 62 plans also had a failing IC measurement. Any plans that could not be measured in their entirety without a shift of the ArcCHECK phantom were excluded from the study. No other plans were excluded. The predominant treatments sites were head and neck (366 plans), brain (82 plans), liver (50 plans), and lung (47 plans). The remaining 166 plans included various other treatment sites. All plans were optimized with version 11 of the Eclipse treatment planning system using the AAA beam model.

## III. RESULTS

The complexity script was used to retrospectively calculate the complexity metric in a batch processing mode for the 711 treatment plans. For several categories, Table 1 shows the number of plans that failed QA, the number of plans above our chosen complexity threshold of $0.18\;{\text{mm}}^{-1}$, and the intersection of these two sets. The mean and standard deviation of the complexity of passing and failing plans is also shown. Brain, lung, and liver body sites are broken into standard fractionation and SBRT fractionation.

**Table 1:** Complexity statistics for all plans analyzed and the four most common VMAT treatment sites

*Body Site*	*Total Plans*	*Failing Plans*	*Plans Above Complexity Threshold*	*Failing Plans Above Complexity Threshold*	*Mean Complexity of Passing Plans* $(mm^{-1})\pm SD$	*Mean Complexity of Failing Plans* $(mm^{-1})\pm SD$
All	711	62	75	27	0.132±0.036	0.170±0.040
Head and Neck	366	25	31	8	0.141±0.030	0.167±0.026
Brain	63	12	8	5	0.117±0.032	0.172±0.053
Brain SBRT	19	7	12	7	0.176±0.037	0.216±0.029
Liver	22	1	1	0	0.129±0.049	0.159
Liver SBRT	28	2	4	0	0.130±0.046	0.145±0.019
Lung	37	2	1	0	0.115±0.032	0.146±0.011
Lung SBRT	10	0	2	0	0.145±0.029	‐

**Figure 3:**
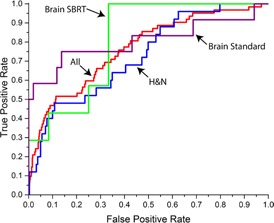
ROC analysis of the ability of the complexity metric to flag plans that failed pretreatment QA. The ROC curves are broken into different body sites: all sites (red), head and neck (blue), standard fractionation brain (purple), and hypofractionated brain (green).

Figure 3 shows an ROC curve generated by varying the complexity metric threshold and plotting the true‐positive rate vs. the false‐positive rate for all sites, as well as for head and neck, standard fractionation brain, and hypofractionated brain. Considering all sites, we found that with a threshold value of $0.18\;{\text{mm}}^{-1}$, the complexity metric correctly flagged 44% of plans that failed pretreatment QA (27 of 62) while incorrectly flagging 7% of plans that passed pretreatment QA (48 of 649).

## IV. DISCUSSION

In this study, we have demonstrated a correlation between plan aperture complexity and VMAT plan deliverability. Using the complexity metric during planning has the potential to save clinical effort, improves overall plan quality, and directly benefits patients. Time savings could come in the form of reduced replans due to failing QA, as well as a reduced number of plans being rejected during the physics plan check due to overmodulation.

Figure 2 shows histograms of the distribution of complexities for all plans and head and neck plans alone. The histograms illustrate that plan complexity can span a wide range (over nearly a factor of ten). Head and neck plans tend to be more complex than the average plan. This large range of complexity values may be due to a number of reasons, including varying patient geometries, clinical priorities (e.g., proximity of organs at risk necessitating increased modulation), planner ability, and planner experience changing over time.

Although head and neck plans are more complex on average, they have proportionately fewer plans above the complexity metric threshold than brain treatment plans. As illustrated in Table 1, together, brain and brain SBRT plans make up only 12% of the sample but 31% of the failing cases. Head and neck plans make up 51% of the sample but only 40% of the failing cases. This corresponds with the fact 24% of brain plans are over the complexity metric threshold while only 9% of head and neck plans are. Brain plans often involve small targets and sometimes multiple spatially separated targets. This type of treatment geometry both tends to increase the complexity metric value and simultaneously push the limits of the dose calculation algorithm.

Figure 3, which shows the ROC curve for all sites, as well as head and neck, brain, and brain SBRT, illustrates the fact that the predictive power of the complexity metric varies for different body sites. This suggests that the volume and geometry of the target and its relation to nearby OARs could be taken into account more directly within the metric to allow an improved correlation between the metric and the delivery accuracy. Another option to take into account the treatment geometry would be to vary the allowed complexity threshold for different body sites. More statistics are needed for other body sites besides head and neck to determine if this approach would be successful. An additional reason to vary the allowed threshold between body sites would be the proximity of neighboring OARs. In certain cases, the planning goals may necessitate a higher degree of plan complexity and delivery uncertainty. At this time, the final assessment of the delivery uncertainty is still measurement‐based QA.

The complexity metric implemented here is simple enough that it could be extended to other planning systems and could be implemented as part of the planning and QA process in other clinics. It is expected that the range of complexity values observed at other institutions would be similar to those shown here, with potentially larger variations between planning systems due to differences in optimization techniques, as well as beam and MLC modeling. Clinics with less experience with VMAT planning may see higher complexity values, as we did when our institution began treating patients with VMAT in 2013. Our results show that using a complexity threshold of $0.18\;{\text{mm}}^{-1}$ may prevent 44% of QA failures with a 7% false‐positive rate during treatment planning. A complexity threshold value is necessary for the type of analysis shown in this work. We chose our threshold such that we could achieve a false‐positive rate of less than 10%. However, in clinical practice it may also make sense to have a “soft” threshold and report the likelihood that a plan will pass or fail QA based on its calculated complexity and the performance of past plans. The appropriate complexity metric threshold may change based on the accuracy of an institution's dose calculation algorithm, how well the clinical data are modeled, the chosen VMAT QA criteria, and the ability of the institution's treatment machine(s) to faithfully deliver the planned treatment. Additionally, adjusting the form of the metric itself may help improve the correlation between the metric value and the delivery accuracy depending on the specific strengths and weaknesses of an institution's dose calculation algorithm.^(9)^ It is also likely that the form of the metric that works best as a penalty during optimization is not the same as that which works best when analyzing complexity postoptimization. This is a topic we plan to investigate further in the future.

This work complements other studies that have investigated the relationship of plan deliverability with treatment plan parameters. Götstedt et al.^(8)^ measured individual aperture calculation accuracy using EPID and film, and compared it to several metrics, showing that there was a strong positive correlation between calculation accuracy and some of the metrics. McNiven et al.^(6)^ used the modulation complexity score (MCS) to quantify IMRT plan complexity and showed a correlation with delivery accuracy using a 2D diode array for measurement. In our previous work,^(9)^ we used film to show the improved deliverability achievable with simpler plans, both on a per‐aperture basis, as well as for the composite delivery. Other groups have shown that commonly used quantitative QA analysis tools and criteria cannot always accurately describe delivery accuracy, especially in the case of highly modulated plans.^13^, ^14^ In this work, when we increased our QA agreement criteria to the more common 3%/3 mm gamma, the number of failing plans decreased from 62 to 29, and the correlation between the complexity metric and QA performance was significantly reduced. Conventional measurement QA is performed using a phantom with very different geometry and heterogeneity characteristics compared to the patient on whom the plan is designed to be delivered. Unless film or another high‐resolution medium is used, measurement points are spaced much farther apart than the calculation points of the plan. All of these factors are reasons why typical QA of inversely optimized treatment plans may not be able to sufficiently describe deliverability.

In this study, while nearly half of QA failures could have been reoptimized before measurement QA, there was still a substantial number of false‐positives. In absolute numbers, there were more false‐positives (48) than true‐positives (27) because there were many more plans passing QA (649) than failing (62). The falsely flagged plans pass pretreatment QA; however, it is important to note that these plans are still the most highly modulated VMAT plans produced in the clinic, and there are many other motivations for reducing complexity beyond ensuring that the plan passes QA. Using the metric as an indicator of overmodulation greatly simplifies the process of determining when reoptimization should be attempted before delivery QA. Even if the plan is below the complexity threshold, if the calculated complexity is far higher than the average for a particular body site, another attempt at optimization is important and can usually be completed quickly. A false‐positive which causes a plan reoptimization that may not have been necessary is much less of a time sink than replanning after failed pretreatment QA.

At our institution, the complexity metric script is used during planning as well as during the physics check of plan quality. Both novice and experienced planners can use the metric to see how their current plan compares to previous plans. If a plan complexity metric is above the threshold value or if the plan is far to the right on the histogram of previous plans (as in Fig. 2), the plan should be reoptimized, unless there is a clear reason for this increased complexity compared to other plans for the same body site. Although no simple, straightforward method of reducing complexity exists, this can sometimes be achieved by small adjustments to the optimization cost function. Complexity often results from unrealistic optimization goals or conflicting optimization objectives. For example, extreme weights on the normal tissue objective for a seemingly simple geometry can often result in very complex plans. Changing the delivery geometry (e.g., adding an additional treatment arc) may also help. However, as suggested above, high complexity is sometimes needed in order to meet the physician‐defined planning goals when the target is irregularly shaped or in close proximity to one or more organs at risk. In these cases, a compromise is necessary between the desired dose distribution and the acceptable level of plan modulation.

In the future, a considerable benefit could be achieved by including the control point complexity in the optimization cost function instead of only restricting MLC motion to meet machine constraints, as is currently done in commercial VMAT solutions. This is akin to including smoothing in IMRT optimization^(15)^ and would allow the planner to more easily find a compromise between meeting plan objectives and achieving the desired level of modulation. In our previous work, we showed that plan complexity could be significantly reduced with very little corresponding change in plan quality.^(9)^ The appropriate use of this type of feature would need to be ensured through a full commissioning by each institution for each dose calculation algorithm used during optimization.

Finally, this type of complexity analysis paves the way for more robust treatment planning solutions that may be less dependent on a measurement pre‐treatment. There has been an ongoing debate on the benefits of pretreatment VMAT and IMRT measurement QA.^16^, ^17^ Without better planning tools, we will continue to remain dependent on these time‐consuming QA methods. Finding a way to ensure that planning systems only generate plans that can be accurately delivered would constitute a major step forward in the goal of streamlining the treatment of patients with inversely optimized treatment plans.

## V. CONCLUSION

Automated analysis of VMAT plan complexity streamlines the VMAT planning process and can improve plan quality. A complexity threshold of $0.18\;{\text{mm}}^{-1}$ resulted in a 44% true positive rate and a 7% false‐positive rate for predicting QA failure. While some highly modulated plans do pass pretreatment QA, there are two important points to remember: not all clinically significant delivery errors are caught with measurement QA, and a high degree of modulation may not be required to meet planning goals. This work is a step along the chain for ensuring high‐quality treatments for all patients, and tools such as the complexity metric will be invaluable for altering our current quality assurance paradigm.

## ACKNOWLEDGMENTS

We wish to thank Dr. Matthew Schipper for his assistance with our ROC analysis. This work was partially funded by NIH P01CA059827.

## COPYRIGHT

This work is licensed under a Creative Commons Attribution 3.0 Unported License.

## Supporting information

Supplementary Material
