# Supplementary figures and images for: Predicting deliverability of volumetric‐modulated arc therapy (VMAT) plans using aperture complexity analysis
Source: J Appl Clin Med Phys. 2016 Jul 8;17(4):124–31. doi: 10.1120/jacmp.v17i4.6241 (PMC5345484; doi:10.1120/jacmp.v17i4.6241)

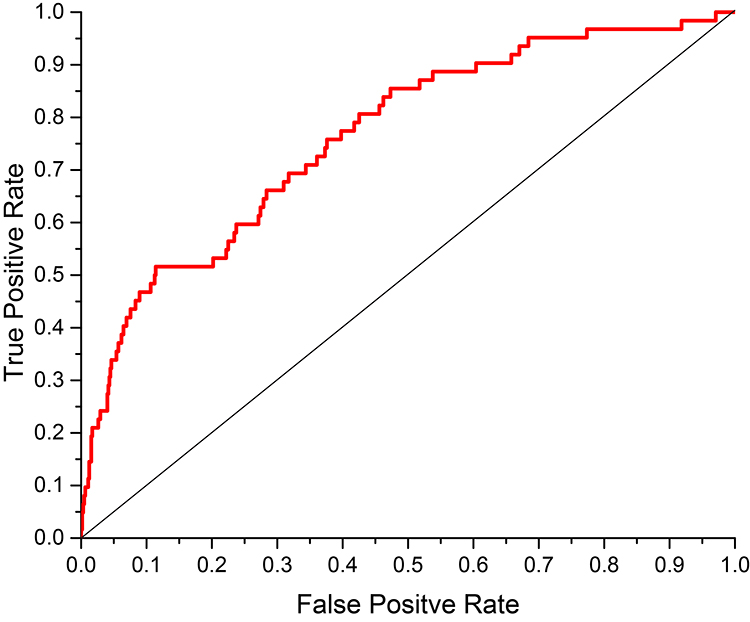

Supplement: Supplementary file 1 — Supplementary Material [file ACM2-17-124-s001.jpg]
